# Climate change effects on Chikungunya transmission in Europe: geospatial analysis of vector’s climatic suitability and virus’ temperature requirements

**DOI:** 10.1186/1476-072X-12-51

**Published:** 2013-11-12

**Authors:** Dominik Fischer, Stephanie M Thomas, Jonathan E Suk, Bertrand Sudre, Andrea Hess, Nils B Tjaden, Carl Beierkuhnlein, Jan C Semenza

**Affiliations:** 1 Department of Biogeography, University of Bayreuth, Bayreuth, Germany; 2 Present address: Technische Universität München (TUM), Munich, Germany; 3 European Centre for Disease Prevention and Control (ECDC), Stockholm, Sweden

**Keywords:** Asian tiger mosquito, Chikungunya, Climate change, Dengue, Globalisation, Global warming, Infectious disease, Invasion, Public health, Vector-borne disease

## Abstract

**Background:**

Chikungunya was, from the European perspective, considered to be a travel-related tropical mosquito-borne disease prior to the first European outbreak in Northern Italy in 2007. This was followed by cases of autochthonous transmission reported in South-eastern France in 2010. Both events occurred after the introduction, establishment and expansion of the Chikungunya-competent and highly invasive disease vector *Aedes albopictus* (Asian tiger mosquito) in Europe. In order to assess whether these outbreaks are indicative of the beginning of a trend or one-off events, there is a need to further examine the factors driving the potential transmission of Chikungunya in Europe. The climatic suitability, both now and in the future, is an essential starting point for such an analysis.

**Methods:**

The climatic suitability for Chikungunya outbreaks was determined by using bioclimatic factors that influence, both vector and, pathogen. Climatic suitability for the European distribution of the vector *Aedes albopictus* was based upon previous correlative environmental niche models. Climatic risk classes were derived by combining climatic suitability for the vector with known temperature requirements for pathogen transmission, obtained from outbreak regions. In addition, the longest potential intra-annual season for Chikungunya transmission was estimated for regions with expected vector occurrences.

In order to analyse spatio-temporal trends for risk exposure and season of transmission in Europe, climate change impacts are projected for three time-frames (2011–2040, 2041–2070 and 2071–2100) and two climate scenarios (A1B and B1) from the Intergovernmental Panel on Climate Change (IPCC). These climatic projections are based on regional climate model COSMO-CLM, which builds on the global model ECHAM5.

**Results:**

European areas with current and future climatic suitability of Chikungunya transmission are identified. An increase in risk is projected for Western Europe (e.g. France and Benelux-States) in the first half of the 21st century and from mid-century onwards for central parts of Europe (e.g. Germany). Interestingly, the southernmost parts of Europe do not generally provide suitable conditions in these projections. Nevertheless, many Mediterranean regions will persist to be climatically suitable for transmission. Overall, the highest risk of transmission by the end of the 21st century was projected for France, Northern Italy and the Pannonian Basin (East-Central Europe). This general tendency is depicted in both, the A1B and B1 climate change scenarios.

**Conclusion:**

In order to guide preparedness for further outbreaks, it is crucial to anticipate risk as to identify areas where specific public health measures, such as surveillance and vector control, can be implemented. However, public health practitioners need to be aware that climate is only one factor driving the transmission of vector-borne disease.

## Background

Chikungunya virus (CHIKV) is an alphavirus (family *Togaviridae*) and was first isolated during an outbreak in Tanzania in 1953 [1]. The virus causes a disease form that typically consists of an acute illness with fever, rash and long-lasting incapacitating arthralgia [2,3]. In recent years, CHIKV has re-emerged in Africa, the Indian Ocean islands (especially on Reunion Island) and the Indian subcontinent as well as South-eastern Asia [3]. The main disease vectors are the two aedine mosquito species, *Aedes aegypti* and *Aedes albopictus *[2,4,5]. In the past, large epidemics were related to the presence of the primary vector *A. aegypti*, the Yellow fever mosquito, which is also the main vector of the dengue virus [2,6,7]. *A. aegypti* was established in southern parts of continental Europe until the mid-1900s but subsequently disappeared for reasons that are not completely understood [7].

During the last few years, *A. aegypti* has established a permanent population in Madeira, Portugal [8], where a recent dengue outbreak occurred [9]. *A. aegypti* has also re-established in the Caucasian region, bordering the Black Sea [10]. It was also introduced further north, such as around the harbour of Rotterdam, Netherlands, in 2010, but mosquito control activities resulted in its eradication in that area [11]. Indeed, establishment of *A. aegypti* might be more difficult in colder climates, as this appears to be a limiting factor for the mosquito in continental Europe [12].

Similarly, temperate regions have proven, thus far, to be of limited suitability for autochthonous CHIKV transmission. The disease was predominantly perceived as travel-related risk in continental Europe until the outbreaks of 2005 and 2006, in which Reunion Island and several neighbouring islands in the Indian Ocean were affected, raising concerns about novel trends of the CHIKV transmission cycle. During this time, genomic micro-evolution of CHIKV enabled transmission by a secondary mosquito vector, *A. albopictus *[4], with the consequence that Chikungunya epidemics can now also occur in regions where the primary vector, *A. aegypti*, is missing [4,5].

The possibility of transmission of CHIKV by *A. albopictus* is significant for continental Europe due to the anthropogenically-faciliated expansion of this mosquito [6,13]. The first introduction of *A. albopictus* in Europe took place in Albania in 1979 [14] and later into the port town of Genoa, Italy, in 1990 due to the importation of used tires [15]. Upon its second arrival, *A. albopictus* became well established in Southern Europe [16,17]. This increases the risk that autochthonous CHIKV transmission may arise, as European populations of *A. albopictus* exhibit a remarkable high vector competence for CHIKV [18,19]. Indeed, the first epidemic of Chikungunya fever in Europe occurred in Ravenna, Northern Italy, with more than 200 affected humans after virus introduction from India [20]. Very recently, two children without travel history became infected in Provence-Alpes-Côte d’Azur, South-eastern France, all originating from a travel-related case coming from an outbreak area in India [21]. In both, the Italian and French outbreaks, *A. albopictus* is believed to have acted as the vector. In another example, *A. albopictus* transmitted dengue virus in Southern France [22] and Croatia in 2010 [23,24]. In light of such developments, along with intensive exchange of travellers between epidemic areas and Europe, the European Centre for Disease Prevention and Control (ECDC) launched a project to assess the risk of introduction and transmission of CHIKV in Europe [25].

Several studies have previously highlighted the increasing climatic suitability for *A. albopictus* in Europe as a consequence of climate change [12,26-28]. Until now, however, the risk for CHIKV transmission has been deduced from the current climatic situation [29]. Climatic requirements for pathogen circulation in outbreak regions and vector suitability must then be addressed as crucial factors [30]. Surprisingly few studies evaluate the spatio-temporal future trends in the risk of CHIKV transmission under European climate change scenarios through the 21st century. Here, we close this research gap by pursuing the following questions:

I. Which continental European regions are at risk (currently and under climate change scenarios), based upon temperature conditions from endemic Chikungunya areas?

II. Which continental European regions are at risk (currently and under climate change scenarios), when accounting for temperature requirements for CHIKV replication and the climatic suitability (including temperature and precipitation) of the vector *A. albopictus*?

III. How long would the potential season of CHIKV transmission last in European areas with assumed *A. albopictus* establishment?

## Methods

### Methodological challenge and strategy in brief

The focus of this study was to determine spatial and temporal climate-derived risk exposure for European regions facing potential transmission of CHIKV. Temperature requirements were derived from the literature based on areas where CHIKV circulated during past outbreaks (1995–2007). These temperature requirements were then used to model the current and expected future climatic suitability for CHIKV transmission in continental Europe.

The climatic suitability for CHIKV transmission was then combined with the climatic suitability of habitats for the vector *A. albopictus* that is based on temperature and precipitation requirements. This was done in order to determine climatic risk classes of CHIKV transmission for European regions by considering both pathogen and vector requirements. In addition, the longest potential intra-annual season for CHIKV transmission was estimated for regions, where vector occurrence has been observed or can be expected in the future. Differences between future projections are evaluated. All analyses were carried out in ArcGIS 10.0™.

### Mapping temperature requirements for the Chikungunya virus

Temperature requirements for CHIKV were obtained from a previous study [29]. Tilston et al. [29] examined progression of several Chikungunya epidemics in relation to local monthly mean temperatures (T_mean_) and derived minimum T_mean_ needed for an outbreak. Interestingly, outbreaks started at different T_mean_ in different geographical localities: 20°C in Italy and Reunion Island, 22°C in India, 24°C in Africa, and 26°C in (Southeast) Asia, respectively.

One conclusion from this is that a T_mean_ of 20°C appears to be the minimum threshold for Chikungunya outbreaks. However, in Italy and Reunion Island, T_mean_ at the beginning of the outbreak was at least 22°C. Hence, we assume a higher CHIKV transmission risk in regions with mean temperatures greater than 20°C for a period of at least one month. This assumption is supported by the fact that an amplification of CHIKV within the vector *A. albopictus* may occur if at least seven days provide temperatures of 26°C [31]. Therefore, higher temperatures will likely increase the risk of transmission as they lead to shorter Extrinsic Incubation Periods (EIP), defined as the time interval between acquisition of an infectious agent by a vector and the vector’s ability to transmit the agent to other susceptible vertebrate hosts.

In order to produce the analysis, the first step involved working with rastered data for T_mean_ for the current situation in Europe, obtained from worldclim.org [32]. For each raster cell, T_mean_ of the warmest month was selected and classified according to the requirements noted above. Projected data for future development of T_mean_ was obtained from the regional climate model COSMO-CLM [33] and classified same way. Pre-processing of the netCDF (network Common Data Form) files of COSMO-CLM demands climate data operator codes [34], before transformation into raster format suitable for a Geographical Information System (GIS) was possible. Spatial resolution of the latter was lower, so climatic data coming from worldclim.org [32] was up-scaled to the 18 km resolution of the COSMO-CLM data via cubic convolution. The COSMO-CLM regional climate model is derived from the driving global model ECHAM5 by dynamical downscaling procedures and covers continental Europe in its entirety [33]. The advantage of working with regional climate models is that they simulate climate change effects more precisely than global models do (resolution > 100 km), which is especially useful for climate change impact studies of ecological processes and vector-borne diseases [35].

Two of the emission scenarios implemented in COSMO-CLM (A1B and B1) were used for climate projections assessment. The A1 scenario family is based on the assumption of an integrated world with single scenarios being characterised by rapid economic growth and a quick spread of new and efficient technologies. Human energy use in the A1B scenario itself is based on a balanced emphasis on all energy sources [36]. The B1 scenario also assumes a globalised world with rapid economic growth, but with changes towards an economy primarily based on service and information. The emphasis is global solutions to economic, social and environmental stability [36]. The B1 scenario corresponds with the ambitious target of the European Union of keeping anthropogenic warming below 2 Kelvin up to the end of the 21st century in comparison to a baseline preindustrial level [37]. To derive climatic trends, T_mean_ data were averaged over intervals of thirty years (2011–2040, 2041–2070, 2071–2100).

### Climatic suitability for the vector *Aedes albopictus*

Data concerning climatic suitability for the vector *A. albopictus* for current and future conditions in Europe was obtained from a previous study [27]. For the purpose of this study, the Maximum Entropy approach, implemented in the MaxEnt software (latest version 3.3.3k) [38], was applied as correlative species distribution model. MaxEnt does not work with real absences, but with an “environmental background”. This approach accounts for both types of “absence” information: either the species does not occur at a given location, or no one has been tried to find it there.

We used the results from the global Statistic-based model (SBM). In short, from a global database of 6347 documented occurrence records of *A. albopictus* a stratified subsampling was conducted resulting in 1119 records that were used as model input in order to avoid inflated results (see [27] for details). Multiple records within one grid cell were additionally removed. The importance of each variable was quantified in a twofold manner with a Jackknife test implemented in MaxEnt. First, models training gain was measured for all variables in isolation and for the remaining set of variables when the isolated variable is dropped from the set. The gain indicates how closely the model is concentrated around the presence samples and can be compared with deviance as a measure of goodness of fit [38]. To reduce collinearity in the data set, variables that had a Pearson correlation coefficient r > 0.7 with any other higher-ranking variable in the results of the Jackknife test were removed. Variables were tested for collinearity before and after upscaling of the climatic data from worldclim.org [32] to the resolution of the COSMO-CLM data. The final input variables of the model are annual mean temperature, annual precipitation, and precipitation of the warmest as well as of the coldest quarter and altitude. Models were trained using a random subset (70%) of occurrence data, tested on the remaining 30% and procedure, replicated 100 times and finally averaged (see [27] for details). The model performance was quantified using the area under the receiver operator characteristic curve (AUC).

The study outputs are climate suitability maps with values ranging from 0 (completely unsuitable) to 1 (extremely favourable conditions). For this study, climate suitability maps were reclassified into five probability classes in equal breaks from zero to one (0.2, 0.4, 0.6, 0.8). Projections of climatic suitability refer to data from the climate model COSMO-CLM [33], time-frames (2011–2040, 2041–2070, 2071–2100) and scenarios (A1B and B1), which were used for projections of CHIKV temperature requirements.

### Risk classification for potential Chikungunya transmission

In order to address the second research question (which regions are at risk and will be at risk under climate change scenarios), pathogen temperature requirements and vector climatic suitability were combined via an overlay procedure. This type of risk classification to detect transmission potential of a vector-borne disease embedded in a GIS environment has been described previously [39].

**Figure 1:**
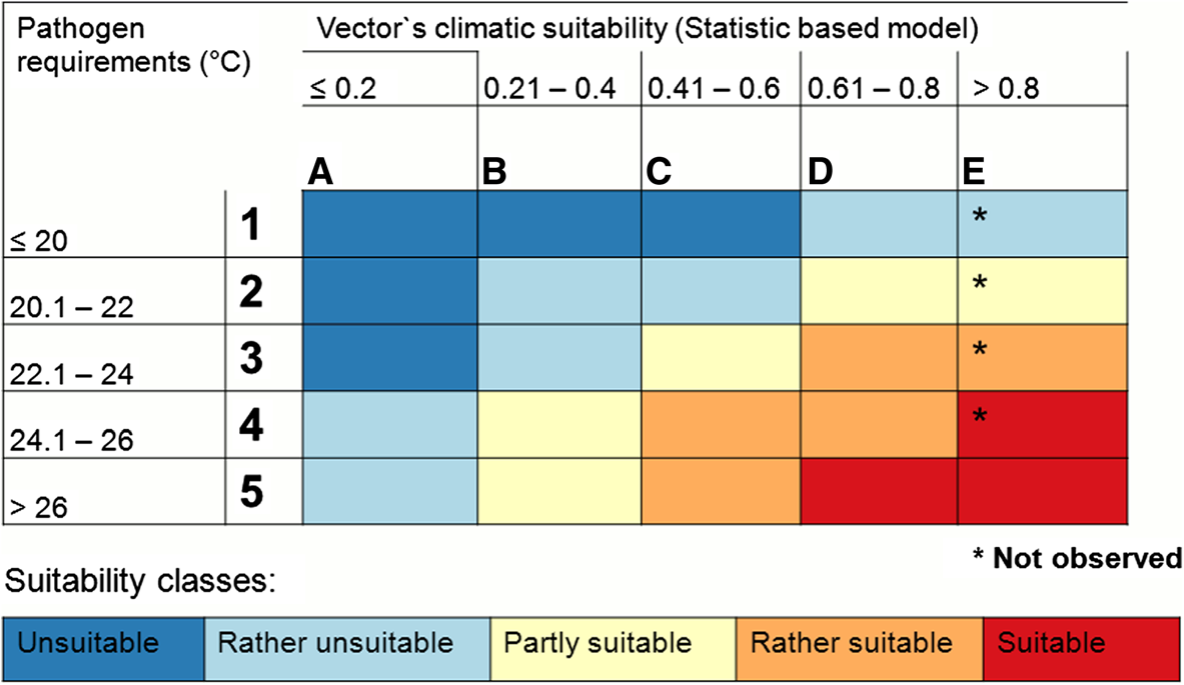
**Climatic-derived risk classes for Chikungunya transmission.** Temperature requirements for the occurrence of Chikungunya virus were obtained from the analysis of Tilston et al. [29]. Chikungunya virus occurrences are observed for values of the mean monthly temperature in different regions. Virus information is combined with the spatial climatic suitability of the vector *Aedes albopictus* from Fischer et al. [27].

We postulate the simple relationship that higher temperatures for the virus and higher climatic suitability for the vector result in higher risk for CHIKV transmission in European regions (Figure 1). Based on this, we created five climatic-derived risk classes upon values for T_mean_, representing pathogens constraints at different geographical regions and five suitability classes for the vector *A. albopictus*. Projections were done for each climate change time-frame and scenario. As precipitation was a variable in the analysis of the vector climate suitability, this ensured that misleading projections of high-risk areas in hot but dry areas are excluded. The results from this overlay were mapped to illustrate risk of CHIKV transmission in Europe, using the raster calculator implemented in ArcGIS 10.0™. Additionally, we calculated the percentage of affected area of each risk class for specific European countries.

### Determining the length of season for Chikungunya transmission

The potential length of the intra-annual season of CHIKV transmission was determined by tallying the number of months in which thermal virus´ requirements are fulfilled for each cell of the environmental raster. In order to gain the most conservative estimate, the threshold was set to a minimum T_mean_ of 20°C (minimum temperature where transmission has been observed according to Tilston et al. [29]). The procedure was carried out for current climatic conditions, each time-frame (2011–2040, 2041–2070, 2071–2100) and scenario (A1B and B1). Once this information was obtained, the number of months with respective minimum T_mean_ (≥ 20°C) were displayed as raster maps.

However, presenting solely number of months suited for pathogen threshold without consideration of potential vector occurrences would overestimate the risk for CHIKV. Consequently, the potential season of transmission was identified for regions with assumed presence of the vector *A. albopictus*. We reclassified the suitability maps of the SBM for the vector *A. albopictus *[27] into binary maps by determining a certain suitability threshold to categorise in regions with expected absence or presence. In environmental niche modelling, a number of procedures for choosing such thresholds exist [40]. Thus, in order to account for the effect of such a threshold choice for species range shifts under climate change [41], we used two established procedures for threshold estimation.

First, a rather classical choice of threshold is separating indices at 0.5, where suitability values range theoretically from zero to one [42,43]. This fixed choice of threshold is not adapted to specific data and modelling results. Second, equalisation of sensitivity and specificity (SeSpeql) by minimising the absolute difference between sensitivity and specificity is another established method [44-47]. Sensitivity and specificity are statistical measures of performance of a binary classification test. Sensitivity measures the proportion of actual positives, which are correctly identified as such. Specificity measures the proportion of negatives which are correctly identified. The probability threshold was chosen at the level where sensitivity (number of true positives divided by the sum of true positives and false negatives) equals specificity (number of true negatives divided by the sum of true negatives and false positives). We calculated the percentage of affected areas for the season of CHIKV transmission for respective European countries (based on SeSpegl-method to determine the threshold of assumed vector occurrence).

## Results

### Temperature requirements for Chikungunya virus

**Figure 2:**
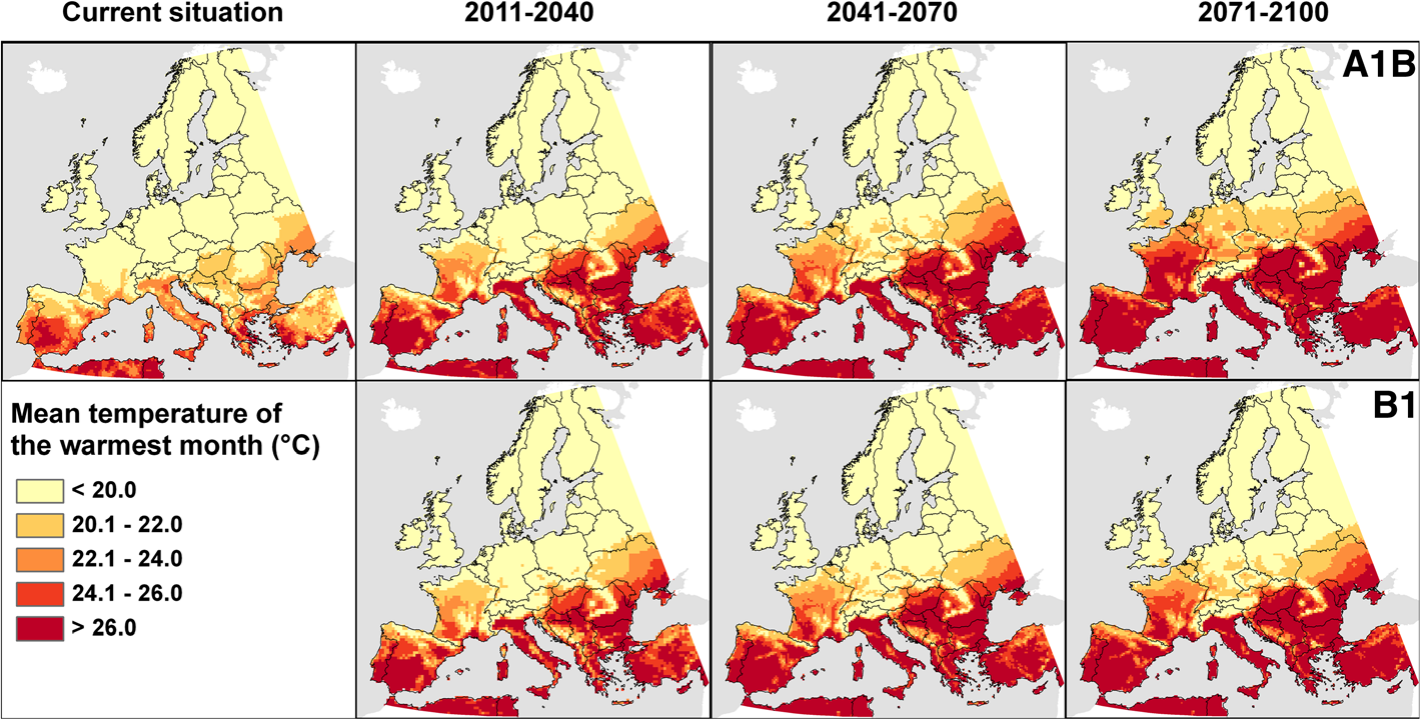
**Fulfilling of temperature requirements for the Chikungunya virus in Europe.** Projections for different time-frames are based on two emission scenarios (A1B and B1) from the Intergovernmental Panel on Climate Change, implemented in the regional climate model COSMO-CLM.

European regions at risk were identified based upon temperature conditions from endemic Chikungunya areas (Figure 2). Based on the previously detected temperature requirements [29], the mean temperature of the warmest month (T_mean_) was mapped for the current situation as well as the projected future (scenarios B1 and A1B, time frames 2011–2040, 2041–2070 and 2071–2100). Currently, western, central, eastern and northern parts of Europe do not have mean temperatures higher than 20°C during the warmest month. Such conditions were solely fulfilled in southern parts of Europe. Generally, large parts of Southern Europe will exceed the lowest observed requirements for T_mean_ of 20°C and achieve values of 26°C. The size of these regions will expand during the 21st century. Interestingly, there are no remarkable differences between the two scenarios concerning temperature conditions for half a century. In the three last decades of the century (2071–2100), in comparison to the B1 scenario, the A1B scenario predicts temperatures of the warmest month to be up to two Kelvin higher in Western, Central and Eastern parts of Europe. This may have severe consequences for Central and Eastern Europe (e.g. Czech Republic, Germany and Poland) as there the lowest requirements were not projected to be fulfilled in the B1 projection while they were for large parts of the countries in the A1B scenario.

### Risk classification by overlaying of vector and virus maps

**Figure 3:**
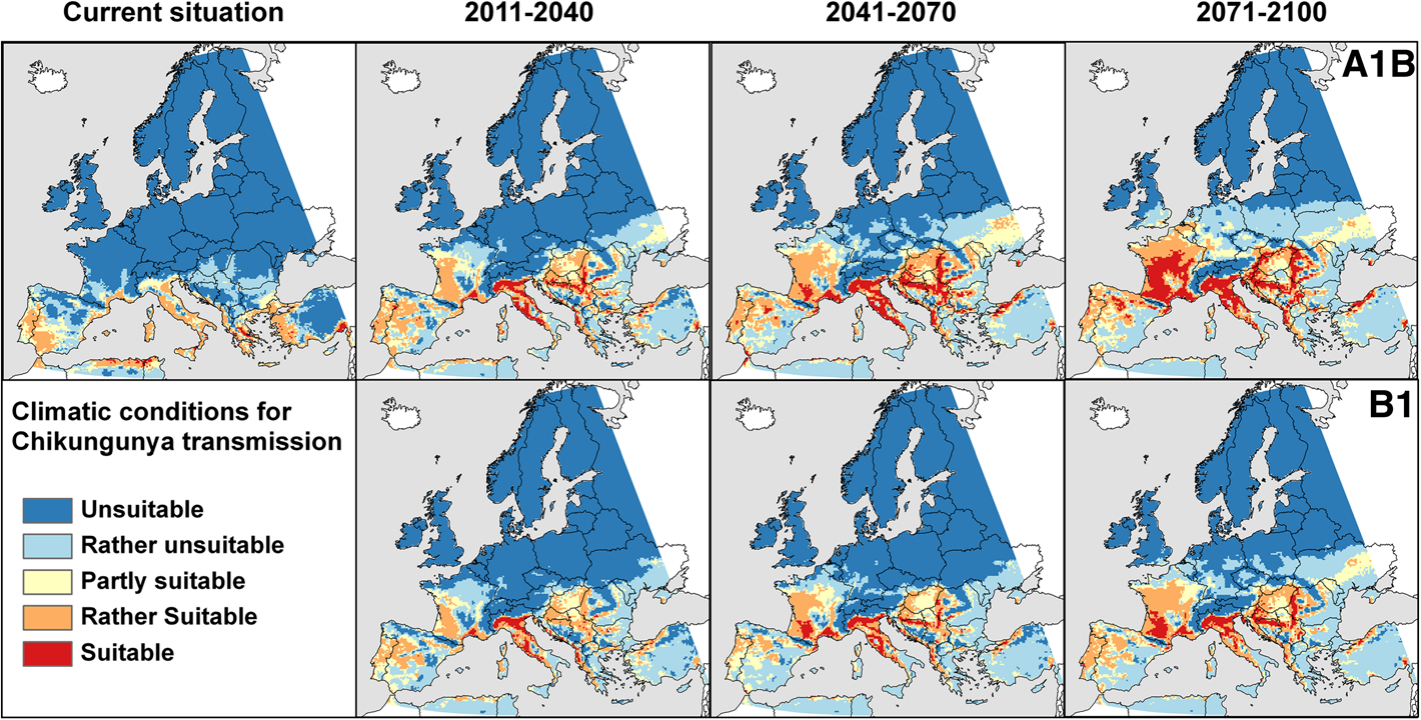
**Risk map for Chikungunya transmission in Europe generated by combining temperature requirements of the Chikungunya virus with the climatic suitability of the vector *Aedes albopictus*.** Projections for different time-frames are based on two emission scenarios (A1B and B1) from the Intergovernmental Panel on Climate Change, implemented in the regional climate model COSMO-CLM.

Assessing which European regions are at risk was done by accounting for temperature requirements for CHIKV replication and the climatic suitability of the vector *A. albopictus*. The models demonstrated high model performance, as indicated by an AUC value of 0.89 (±0.01) for the SBM of the vector. Currently, the risk of CHIKV transmission is highest for the southernmost parts of Europe. As a general tendency, the climatic risk of CHIKV will increase in Europe and the increase in risk exposure is more pronounced in the A1B scenario in comparison to the B1 scenario (Figure 3 and Additional file 1). A persisting high suitability for CHIKV transmission throughout the 21st century is projected for the Po valley in Emilia Romagna, Northern Italy. The climatic risk for CHIKV transmission is moreover projected to increase in the Western coastal Mediterranean areas of the Balkan States and Greece as well as in the Pannonian Basin. The Black Sea coast of Turkey must be aware of increasing climatic suitability for CHIKV transmission.

A spatially limited risk is projected for mid-century conditions in Central Europe. South-eastern parts of the British Isles will be at limited risk at the end of the 21st century, according to both currently available scenarios. The northernmost parts, Scandinavia and the Baltic states, will not likely be subject to climate-induced risk.

### Potential season of transmission

**Figure 4:**
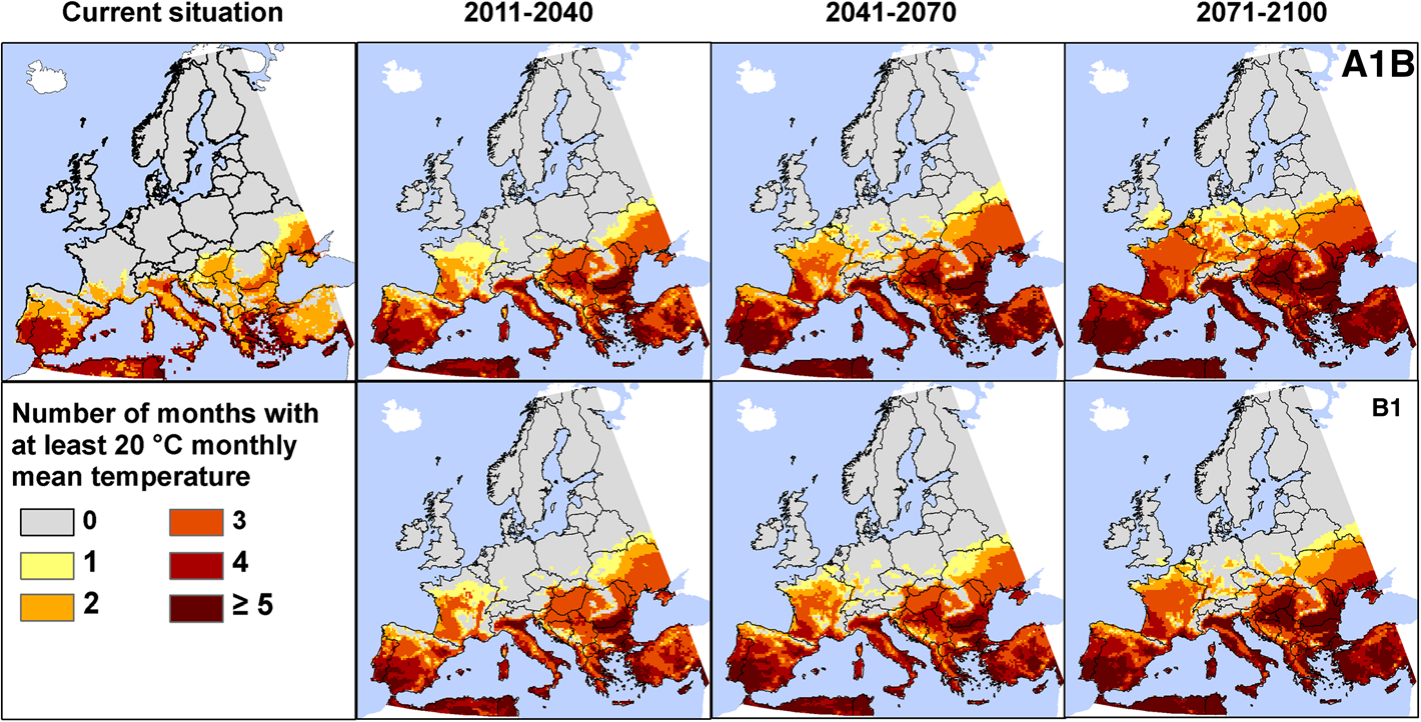
**Number of months with mean temperature ≥20°C as minimum requirement for the transmission of Chikungunya virus.** Projections for different time-frames are based on two emission scenarios (A1B and B1) from the Intergovernmental Panel on Climate Change, implemented in the regional climate model COSMO-CLM.

A final research question for this paper relates to the potential season of CHIKV transmission in Europe. First, we present only the number of months with suitable temperatures for CHIKV replications, without consideration of the vector. Obviously, the number of months with suitable temperatures increases for many European regions (Figure 4 and Additional file 1). Currently, a T_mean_ of 20°C or higher in at least one month is restricted to countries in Southern Europe. Yet, by the end of the current time-frame (2011–2040), up to three months can be expected in Western Europe, regardless the chosen climate change scenario. With temporal delay, temperature requirements will be fulfilled for at least one month in Central (2041–2070) and many parts of Eastern Europe (2071–2100). By the end of the century, five months with minimum temperatures of at least 20°C are projected for many parts of Southern Europe (in both scenarios). Differences in scenarios do, however, arise for the end of the 21st century (2071–2100) in Central and Eastern Europe. In the A1B scenario, most of the regions are identified with at least one month of fulfilled requirements, while in B1 scenario only spatially limited regions are highlighted there. In addition, the risk in the south-easternmost part of the United Kingdom is more extended in A1B projection. The tendencies in Western and Southern Europe are the same throughout the 21st century.

**Figure 5:**
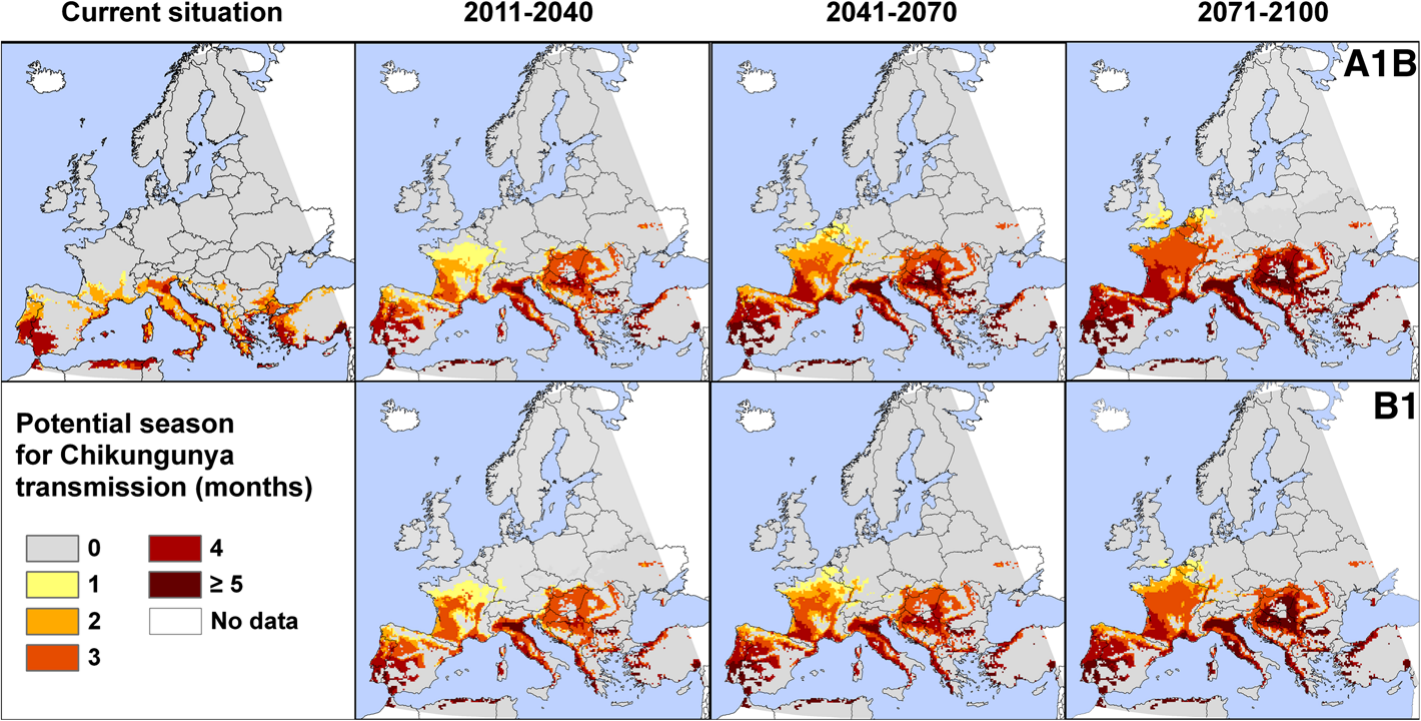
**Length of transmission season for Chikungunya (in months), but filtered by areas, where the presence of the vector *Aedes albopictus *can be expected.** The threshold for occurrences from continuous values of suitability was obtained by minimising the absolute difference between the sensitivity and specificity, resulting in a specific value (0.371) as threshold for vector occurrences. Projections for different time-frames are based on two emission scenarios (A1B and B1) from the Intergovernmental Panel on Climate Change, implemented in the regional climate model COSMO-CLM.

The number of months with suitable temperatures for CHIKV replication is only one part of the story to determine the potential season of transmission. Therefore, we also assessed the number of months with suitable temperatures for CHIVK replications for those regions with expected presence of *A. albopictus*. This determination of the potential transmission season is based on the number of months with the lowest observed temperature requirement of 20°C and on the modelled distribution of the vector *A. albopictus*. The threshold for vector presences via SeSpeql-method was calculated to be 0.371. We also used the classical fixed value of 0.5. Presence of the species can be expected if these thresholds are met or exceeded in the respective region. Due to lower threshold value for the occurrence of *A. albopictus* via SeSpeql-method, more areas are identified where *A. albopictus* may be present, in comparison the conservative fixed threshold of 0.5. This resulted in more regions under consideration for the potential season of transmission by applying the SeSpeql-method (Figure 5) than by applying the conservative threshold (Additional file 2).

The following detailed interpretation is for the results of the SeSpeql-method (Figure 5). Currently, the longest possible period of transmission is identified along the Mediterranean coast line with a maximum of three months. Regardless of the chosen climate change scenario, areas of widened transmission windows will be the Pannonian Basin, the Po Valley, where the season of transmission might even rise up to five months from mid-century onwards. Moreover, three months of transmission will be possible in wide ranges of Central and Western Europe. In large parts of France the potential season of transmission could be four months. Interestingly, some regions where the potential transmission exists are geographically close to regions where the vector *A. albopictus* is not expected to occur. This is especially apparent in south-eastern parts of the Iberian Peninsula.

## Discussion

### Relevance, novelty and uncertainty in modelling approaches

The European-wide projections for *A. albopictus* account for changing patterns of activity phase or/and climatic suitability [12,26-28]. Frequently, spatial risk analyses for CHIKV transmission are based on calculating (and mapping) the basic reproduction number R_0 _[30,47-49]. However, in the case of CHIKV in Europe this type of modelling can be very misleading as one key factor for such models is the vector density, which is not yet known [50]. Therefore, in this study we pursued an alternative approach, in which we indicate regions where the climate is favourable for CHIKV transmission. Combined risk maps for the climatic suitability of the vector (*A. albopictus*) and the temperature thresholds for CHIKV transmission are derived via classification functions in order to identify spatial patterns at different future time-frames. This insight guided the climatic risk maps presented here of potential CHIKV transmission zones for all of Europe that address both the current and expected future climatic conditions. We provide an additional map indicating all of the mentioned regions and localities of the mentioned European regions or localities in the text for an easy interpretation (Additional file 3). Differences in projected time-frames and scenarios are evaluated. As European climates become more permissive in the future, further spread of *A. albopictus* to higher latitudes on the continental European scale [12,26-28] and to higher altitudes on the local scale is anticipated [51,52].

The objective of this study was to further assess the potential role of climate in CHIKV transmission in continental Europe, both now and in the future. For this purpose, we applied climate change data from a regional climate model in order to to ensure the best accuracy available [35]. One of the main benefits of this study is the consideration of different temperature requirements for Chikungunya outbreaks during current conditions and at different time horizons. Additionally, the approach followed in this paper allowed for the seasonal (intra-annual) trend in potential transmission to be identified. The model results of this current and future climatic risk analyses by combining habitat suitability for the vector *A. albopictus* in Europe [27] and the temperature requirements for the CHIKV [29] are all based on the same climatic data basis, worldclim.org [32] and COSMO-CLM [33], respectively.

We identify the effect of spatial autocorrelation in MaxEnt models for the vector *A. albopictus* based on *Moran’s I* calculation as a source of uncertainty [see [27]. However, upscaling the bioclimatic data coming from worldclim.org [32] to the resolution of the data from COSMO-CLM [33] had no significant effect. Consistent with the findings of other authors [53], we note that geographical corrections of clustered data improved reliability of predictions due to lower values of *Moran’s I*, but did not resolve the problem entirely.

The choice of a threshold for determining species presence is one of the most challenging issues in species distribution modelling [40]. Reliability of risk analysis for CHIKV transmission that is based on the presence of a competent mosquito vector is highly sensitive to this issue. In order to consider the range of approaches, two established settings were applied. First, we compared the results to those that were derived from a conservative and fixed choice of threshold of 0.5 [42,43]; and second, in order to consider the findings that the general threshold contributed to uncertainty in predictions under climate change [40], data-adapted thresholds choice based on SeSpeql was used that is considered as one of the most accurate threshold choices [54,55]. The results from this study demonstrate that differences concerning vector occurrences are remarkable when applying different threshold criteria. Consequently, interpretation of results is intricate for those regions where the intra-annual season for transmission is determined on the basis of assumed occurrences of the vector *A. albopictus*.

### Climate data: limitations and assumptions for risk analyses

The role of climate change in the recent outbreak of CHIKV in Northern Italy, the first recorded outbreak in a temperate region, is uncertain [56]. The impact of globalisation, however, is clear, as travel and trade lead to the introduction of *A. albopictus* in Europe and the subsequent introduction of CHIKV into a formerly non-endemic area [3]. It should be noted that the future-orientated models in this paper are based upon climatic factors*.* The risk with this approach is that climate-impact studies are inevitably vulnerable to some degree of climate reductionism, in which climatic drivers of change are prioritised vis-à-vis other important disease drivers [57]. One reason for this is that there are no good projections that consider biological, socioeconomic or other drivers of the future spread of CHIKV. In this study, socioeconomic factors are incorporated only through the way in which they factor into the different emissions assumptions underpinning the IPCC A1B and B1 scenarios.

Currently, the next generation of climate scenarios are in development, which will be helpful for future climate impact studies [58]. The new parallel process for the development of such scenarios is characterised by an extensive exchange between scientific disciplines. One major advantage is that socio-economic uncertainties affecting both adaptation and mitigation appear to be better accounted for, perhaps particularly in the rainfall induced climate extremes [58,59]. This becomes even more crucial, as the impact of precipitation on vector distribution is elusive. In general, precipitation signals depend on local phenomena [59], leading to temporary increases of breeding sites for mosquitoes after e.g. heavy rains. Any deviations in the relationship between heavy rains and breeding sites can reasonably be assumed to be caused by human activities [60].

In coastal Kenya, the epidemic Chikungunya fever emergence after unusually warm, dry conditions, whereas previous epidemics in Africa and Asia followed heavy rain [61]. The applied niche model for vector’s potential distribution does also account for a certain amount of rainfall as important explanatory variable in a global dimension. However, reality on the regional or local scale is more complex. In Kenya, infrequent replenishment of water stores during drought may have led to an increase of domestic *A. aegypti* populations, thus heightening the risk for CHIKV circulation. In the Mediterranean an increase of frequency of droughts has already been observed [62]. Here, private water storages may create additional breeding sites for the container breeding mosquitoes in regions where occurrences are not projected yet. However, within this study we do not address local and short-term phenomena but focus on general tendencies in a longer temporal dimension on a continental scale of Europe.

### Vector and pathogen specific factors in disease transmission

One assumption in this study is that evidence from the current climatic situation can help to detect risk zones of vector-borne diseases. However, the vector as well as the virus could evolve to their changing environment in space and/or time, with unpredictable results. In the case of the vector, climatic data were used as explanatory variables of a species distribution model for the vector *A. albopictus *[27]. It is worth mentioning that *A. albopictus* prefers anthropogenic habitats and has further environmental or biological preferences, which are not accounted for in our niche model. Nevertheless, it is shown that climatic-derived distribution models can predict the current distribution of this mosquito in Europe at a high spatial resolution (< 20 km) in a valid quality [26-28].

The risk analysis is exclusively based on one possible vector species, namely *A. albopictus*. In addition to the vector competence of *A. albopictus* for CHIKV, it must be taken into account that further aedine species are also capable of transmitting this alphavirus [2,3]. Biotic interactions e.g. between competitive mosquitoes in larval or adult stage may play a decisive role in species establishment. The primary vector is thought to be *A. aegypti*. The risk of re-establishment of *A. aegypti* in the continental interior of Europe is on one hand related to permanent populations of the mosquito in Madeira [9] and the Caucasian region of the Black Sea [10], and on the other hand to continual introductions by intercontinental transportation. In particular past experiences of the Netherlands showed introduced populations [63] originated from Miami, Florida, USA which are currently eliminated due to intensified mosquito control activities [11]. This highlights the necessity to account for a Europe-wide control of intercontinental transportation systems [64]. In order to detect areas for mosquito control activities, the minimum survival temperature of mosquito eggs over the winter should be taken into account. *A. aegypti* only tolerates long term cold treatments not lower than -2°C; a -7°C cold period for more than one hour causes a complete breakdown of hatching [65]. Therefore, the establishment and spread of *A. aegypti* in temperate Europe seems to be mitigated by European winter temperatures. In any case, *A. albopictus* is probably the mosquito that replaces resident and further invasive mosquito most effectively e.g. [66], justifying the focus on this vector in this study.

The frost tolerance of *A. albopictus* may be crucial for risk analyses. In Italy, cold acclimation as overwintering strategy has been observed for *A. albopictus *[67]. Under laboratory conditions, the low-temperature thresholds for the survival of post-diapausing and non-diapausing eggs of *A. albopictus* have been identified [65]. It can be assumed that besides changes in long-term trends the frequency and intensity of climatic extremes will increase [59], which will have serious effects for the alteration of vector habitats, which has not been accounted so far in projections of distribution.

The temperature required for CHIKV transmission was adapted from the compilation of endemic regions given in Tilston et al. [29]. This contains the risk that temperature requirements used here may be superimposed by other factors, which were not accounted for. A more accurate way to determine a temperature threshold for transmission would be to identify the extrinsic incubation period (EIP) via laboratory experiments [68]. For the dengue virus, this temperature-dependent EIP has been mapped and projected for the European continent [69]. In the case of CHIKV, concrete laboratory controlled studies aiming to determine the temperature-dependent EIP of different CHIKV strains in different vectors are currently missing.

### Outlook and concluding remarks

In general, there is a growing consensus that infectious diseases transmitted via vectors are especially affected by climate change, when regarding the northern limits of distribution [70], which is also shown within this study. Additional work should be conducted to improve the models and/or with laboratory data about the temperature requirement in light of virus evolution and changing vector distribution [71]; it should combine both information on pathogen requirements and bioclimatic conditions of the vector(s) *A. albopictus* and *A. aegypti.* It would be furthermore of particular interest to distinguish between areas of possible establishment of aedine vectors in Europe and areas with sufficiently long weeks of activity (ranging from spring hatching to autumn diapause).

As a consequence of global transport and travelling, several exotic viruses and/or disease vectors were introduced in Europe and became established thereafter [13]. This necessitated vector control strategies [72]. In current years, the number of travel-related CHIKV infections increased in many European countries [73]. Combined assessment of potential virus introduction by using e.g. the VBD-Air tool [74] with climatic zones may form an evidence base for concepts of efficient mitigation strategies.

Once climatic risk zones and potential introduction gateways have been identified, a comprehensive CHIKV risk assessment needs to be expanded to account for societal and demographic drivers in order to adapt public health systems [75,76]. Then, an overall view of all relevant impacts could be used to evaluate the way in which surveillance ought to be implemented or modified [77]. If diseases emerge, then adaptation strategies are required to be available in order to protect public health from the impending threat [78].

## Abbreviations

A. aegypti: *Aedes aegypti* (Yellow fever mosquito); A. albopictus: *Aedes albopictus* (Asian tiger mosquito); AUC: Area under the receiver operator characteristic curve; CHIKV: Chikungunya virus; ECDC: European Centre for Disease Prevention and Control; EIP: Extrinsic incubation period; GIS: Geographical Information System; netCDF: network Common Data Form; SBM: Statistic-based model; SeSpeql: Equalisation of sensitivity and specificity (as threshold-setting method); Tmean: Mean monthly temperature.

## Competing interests

The authors declare that they have no financial or non-financial competing interests.

## Authors’ contributions

JCS, JES and BS initiated the project “Climate modelling for Chikungunya” (OJ/08/02/2012-PROC/2012/012). DF, SMT and CB developed the idea for the specific analysis. DF, AH, SMT and NBT practised the analysis. CB, JCS, JES and BS contributed to the model design. DF, SMT and AH prepared figures and additional files. All authors contributed to the manuscript, commented on drafts critically and edited the final version of this paper. All authors read and approved the final version of the manuscript.

## Supplementary Material

Additional file 1 **Percentage of affected area of France, Germany, Greece, Italy and Spain in the respective risk classes and the potential season of transmission periods for the A1B and B1 scenario during this century according to Figures **3** and **5. The calculation of transmission is based on the method of equalisation of sensitivity and specificity as threshold-setting method to determine vector’s occurrence. Click here for file

Additional file 2 **Potential season of transmission for Chikungunya virus.** From the map of the number of months with at least 20°C as mean temperature, only those areas were considered, where the presence of the vector *Aedes albopictus* can be expected, according to the fixed threshold (0.5) for vector occurrences from the continuous scale for climatic suitability. Projections for different time-frames are based on the two IPCC-scenarios (A1B and B1), implemented in the regional climate model COSMO-CLM. Click here for file

Additional file 3 Map of mentioned European regions and localities in the main text. Click here for file
