# Supplementary material for: Climate change effects on Chikungunya transmission in Europe: geospatial analysis of vector’s climatic suitability and virus’ temperature requirements
Source: Int J Health Geogr. 2013 Nov 12;12:51. doi: 10.1186/1476-072X-12-51 (PMC3834102; doi:10.1186/1476-072X-12-51)
Supplement: Additional file 1 — Percentage of affected area of France, Germany, Greece, Italy and Spain in the respective risk classes and the potential season of transmission periods for the A1B and B1 scenario during this century according to Figures 3 and 5. The calculation of transmission is based on the method of equalisation of sensitivity and specificity as threshold-setting method to determine vector’s occurrence. [file 1476-072X-12-51-S1.doc]

| **A1B Scenario** | **Risk classes (% area)** | | | | | **Potential Season of Transmission (% area)** | | | | | |
| --- | --- | --- | --- | --- | --- | --- | --- | --- | --- | --- | --- |
|  |  | | | | |  | | | | | |
|  | **1** | **2** | **3** | **4** | **5** | **0 month** | **1 month** | **2 months** | **3 months** | **4 months** | **5 months** |
| **France** |  |  |  |  |  |  |  |  |  |  |  |
| Current | 78,71 | 13,89 | 3,33 | 4,07 | 0 | 83,84 | 4,49 | 10,88 | 0,58 | 0,21 | 0 |
| 2011-2040 | 24,76 | 27,35 | 20,54 | 25,55 | 1,8 | 37,17 | 28,09 | 20,17 | 12,14 | 2,43 | 0 |
| 2041-2070 | 9,29 | 20,06 | 20,59 | 42,4 | 7,66 | 24,71 | 5,6 | 33,95 | 20,86 | 14,1 | 0,79 |
| 2071-2100 | 4,17 | 3,75 | 9,87 | 42,82 | 39,39 | 21,7 | 0,05 | 5,6 | 46,52 | 23,97 | 2,16 |
|  | | | | | |  | | | | | |
| **Germany** |  |  |  |  |  |  |  |  |  |  |  |
| Current | 100 | 0 | 0 | 0 | 0 | 100 | 0 | 0 | 0 | 0 | 0 |
| 2011-2040 | 96,23 | 3,77 | 0 | 0 | 0 | 96,63 | 3,29 | 0,08 | 0 | 0 | 0 |
| 2041-2070 | 71,47 | 26,28 | 2,16 | 0,08 | 0 | 89,9 | 4,65 | 5,05 | 0,4 | 0 | 0 |
| 2071-2100 | 14,5 | 59,29 | 22,52 | 3,69 | 0 | 84,29 | 3,53 | 2,32 | 9,86 | 0 | 0 |
|  | | | | | |  | | | | | |
| **Greece** |  |  |  |  |  |  |  |  |  |  |  |
| Current | 5,96 | 9,49 | 31,98 | 43,36 | 9,21 | 31,44 | 0,27 | 26,02 | 5,15 | 36,04 | 1,08 |
| 2011-2040 | 8,38 | 54,05 | 12,16 | 23,51 | 1,89 | 75,14 | 0,27 | 1,89 | 5,68 | 10 | 7,03 |
| 2041-2070 | 1,35 | 61,62 | 14,86 | 17,84 | 4,32 | 72,7 | 0,54 | 0,27 | 4,59 | 6,49 | 15,41 |
| 2071-2100 | 0 | 67,3 | 12,16 | 18,38 | 2,16 | 73,24 | 0 | 0,27 | 1,08 | 7,3 | 18,11 |
|  | | | | | |  | | | | | |
| **Italy** |  |  |  |  |  |  |  |  |  |  |  |
| Current | 19,07 | 12,21 | 25,53 | 42,48 | 0,71 | 32,29 | 1,61 | 29,57 | 13,02 | 23,41 | 0,1 |
| 2011-2040 | 18,87 | 20,48 | 11,5 | 22 | 27,14 | 50,76 | 1,31 | 3,33 | 13,82 | 26,84 | 3,94 |
| 2041-2070 | 16,35 | 24,42 | 7,97 | 16,04 | 35,22 | 48,94 | 0,3 | 1,72 | 9,89 | 19,17 | 19,98 |
| 2071-2100 | 13,22 | 24,82 | 8,17 | 17,05 | 36,73 | 48,54 | 0,2 | 0,5 | 5,45 | 13,22 | 32,09 |
|  | | | | | |  | | | | | |
| **Spain** |  |  |  |  |  |  |  |  |  |  |  |
| Current | 24,49 | 34,18 | 23,33 | 17,93 | 0,06 | 71,33 | 2,15 | 9,17 | 0,41 | 16,95 | 0 |
| 2011-2040 | 12,09 | 32,47 | 26,1 | 28,99 | 0,35 | 53,88 | 0,64 | 10,71 | 5,84 | 16,61 | 12,33 |
| 2041-2070 | 5,44 | 40,34 | 27,89 | 23,67 | 2,66 | 58,97 | 4,4 | 10,42 | 10,24 | 13,37 | 2,6 |
| 2071-2100 | 1,97 | 37,67 | 28,47 | 31,31 | 0,58 | 53,65 | 0 | 2,26 | 5,73 | 22,28 | 16,09 |

| **B1 Scenario** | **Risk classes (% area)** | | | | | **Potential Season of Transmission (% area)** | | | | | |
| --- | --- | --- | --- | --- | --- | --- | --- | --- | --- | --- | --- |
|  |  | | | | |  | | | | | |
|  | **1** | **2** | **3** | **4** | **5** | **0 month** | **1 month** | **2 months** | **3 months** | **4 months** | **5 months** |
| **France** |  |  |  |  |  |  |  |  |  |  |  |
| Current | 29,51 | 34,16 | 16,63 | 18,43 | 1,27 | 38,81 | 24,5 | 10,93 | 24,55 | 1,21 | 0 |
| 2011-2040 | 15,36 | 25,87 | 21,44 | 29,88 | 7,44 | 28,83 | 13,67 | 23,13 | 24,39 | 9,98 | 0 |
| 2041-2070 | 7,29 | 17,21 | 19,96 | 43,98 | 11,56 | 23,23 | 4,22 | 15,58 | 42,77 | 13,25 | 0,95 |
| 2071-2100 | 29,51 | 34,16 | 16,63 | 18,43 | 1,27 | 38,81 | 24,5 | 10,93 | 24,55 | 1,21 | 0 |
|  | | | | | |  | | | | | |
| **Germany** |  |  |  |  |  |  |  |  |  |  |  |
| Current | 96,55 | 3,45 | 0 | 0 | 0 | 96,71 | 3,21 | 0,08 | 0 | 0 | 0 |
| 2011-2040 | 86,06 | 13,3 | 0,64 | 0 | 0 | 91,67 | 5,53 | 2,72 | 0,08 | 0 | 0 |
| 2041-2070 | 65,95 | 30,93 | 3,13 | 0 | 0 | 89,5 | 3,37 | 5,13 | 2 | 0 | 0 |
| 2071-2100 | 96,55 | 3,45 | 0 | 0 | 0 | 96,71 | 3,21 | 0,08 | 0 | 0 | 0 |
|  | | | | | |  | | | | | |
| **Greece** |  |  |  |  |  |  |  |  |  |  |  |
| Current | 9,19 | 58,65 | 11,62 | 20,27 | 0,27 | 72,7 | 0 | 1,89 | 6,76 | 12,7 | 5,95 |
| 2011-2040 | 4,86 | 68,38 | 13,24 | 13,51 | 0 | 74,86 | 0,54 | 0 | 4,86 | 12,7 | 7,03 |
| 2041-2070 | 2,43 | 73,78 | 14,32 | 9,46 | 0 | 74,32 | 0,27 | 0,27 | 3,78 | 5,68 | 15,68 |
| 2071-2100 | 9,19 | 58,65 | 11,62 | 20,27 | 0,27 | 72,7 | 0 | 1,89 | 6,76 | 12,7 | 5,95 |
|  | | | | | |  | | | | | |
| **Italy** |  |  |  |  |  |  |  |  |  |  |  |
| Current | 19,58 | 23,81 | 9,28 | 26,54 | 20,79 | 50,15 | 1,21 | 2,93 | 19,88 | 16,75 | 9,08 |
| 2011-2040 | 17,46 | 27,55 | 8,88 | 23,31 | 22,81 | 49,95 | 0,81 | 1,11 | 13,02 | 27,85 | 7,27 |
| 2041-2070 | 16,25 | 28,05 | 7,87 | 25,33 | 22,5 | 48,64 | 0,4 | 0,81 | 10,19 | 11,3 | 28,66 |
| 2071-2100 | 19,58 | 23,81 | 9,28 | 26,54 | 20,79 | 50,15 | 1,21 | 2,93 | 19,88 | 16,75 | 9,08 |
|  | | | | | |  | | | | | |
| **Spain** |  |  |  |  |  |  |  |  |  |  |  |
| Current | 15,34 | 39,64 | 25,23 | 19,68 | 0,12 | 58,74 | 3,88 | 9,03 | 14,35 | 10,71 | 3,3 |
| 2011-2040 | 8,39 | 51,56 | 26,62 | 12,67 | 0,75 | 56,08 | 2,55 | 7,81 | 10,36 | 15,74 | 7,47 |
| 2041-2070 | 5,67 | 45,08 | 27,08 | 21,47 | 0,69 | 54,69 | 0,52 | 7,06 | 8,91 | 18,34 | 10,47 |
| 2071-2100 | 15,34 | 39,64 | 25,23 | 19,68 | 0,12 | 58,74 | 3,88 | 9,03 | 14,35 | 10,71 | 3,3 |
