# Supplementary figures and images for: Climate change effects on Chikungunya transmission in Europe: geospatial analysis of vector’s climatic suitability and virus’ temperature requirements
Source: Int J Health Geogr. 2013 Nov 12;12:51. doi: 10.1186/1476-072X-12-51 (PMC3834102; doi:10.1186/1476-072X-12-51)

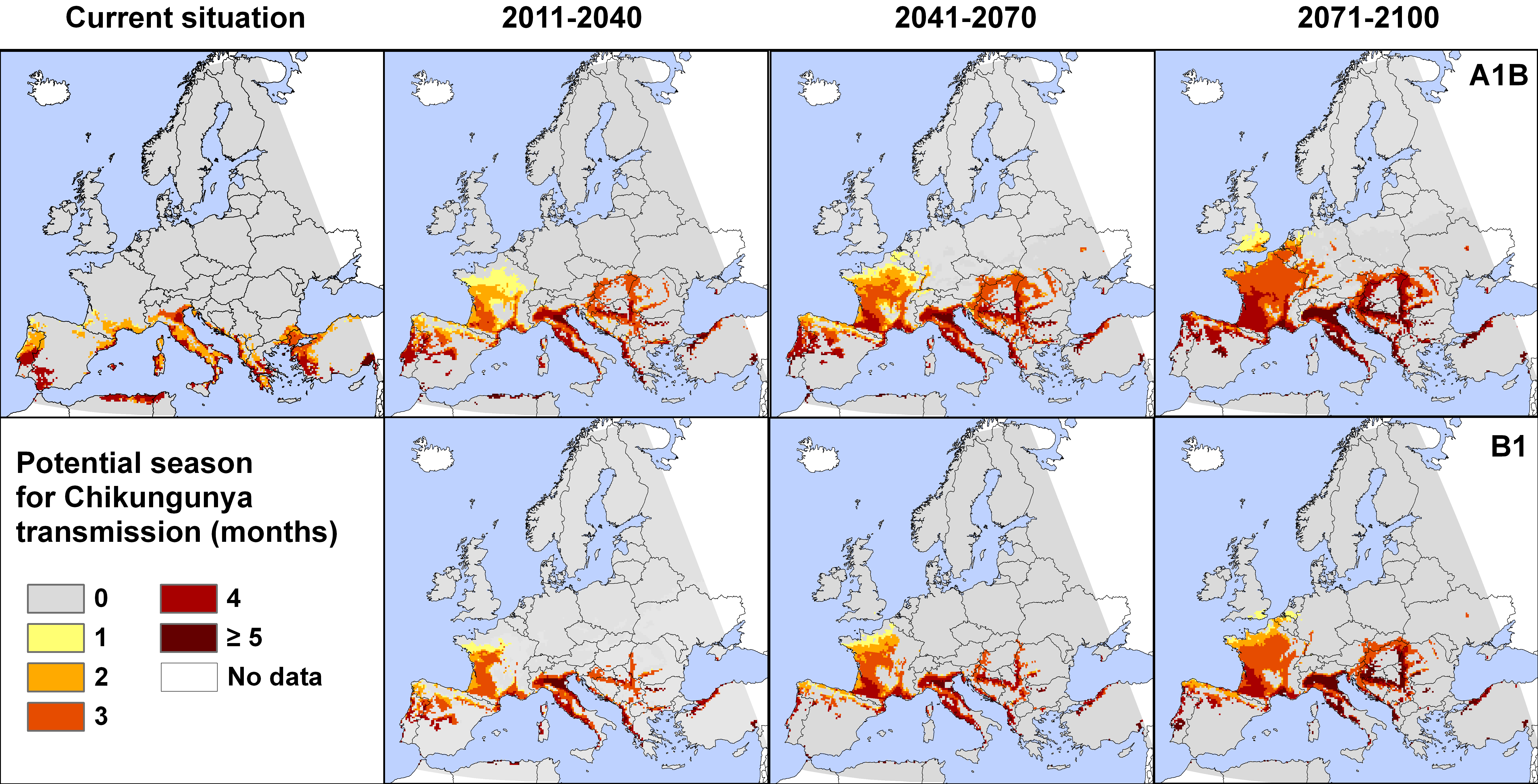

Supplement: Additional file 2 — Potential season of transmission for Chikungunya virus. From the map of the number of months with at least 20°C as mean temperature, only those areas were considered, where the presence of the vector Aedes albopictus can be expected, according to the fixed threshold (0.5) for vector occurrences from the continuous scale for climatic suitability. Projections for different time-frames are based on the two IPCC-scenarios (A1B and B1), implemented in the regional climate model COSMO-CLM. [file 1476-072X-12-51-S2.png]
