# Supplementary material for: Climate change effects on Chikungunya transmission in Europe: geospatial analysis of vector’s climatic suitability and virus’ temperature requirements
Source: Int J Health Geogr. 2013 Nov 12;12:51. doi: 10.1186/1476-072X-12-51 (PMC3834102; doi:10.1186/1476-072X-12-51)
Supplement: Additional file 3 — Map of mentioned European regions and localities in the main text. [file 1476-072X-12-51-S3.pdf]

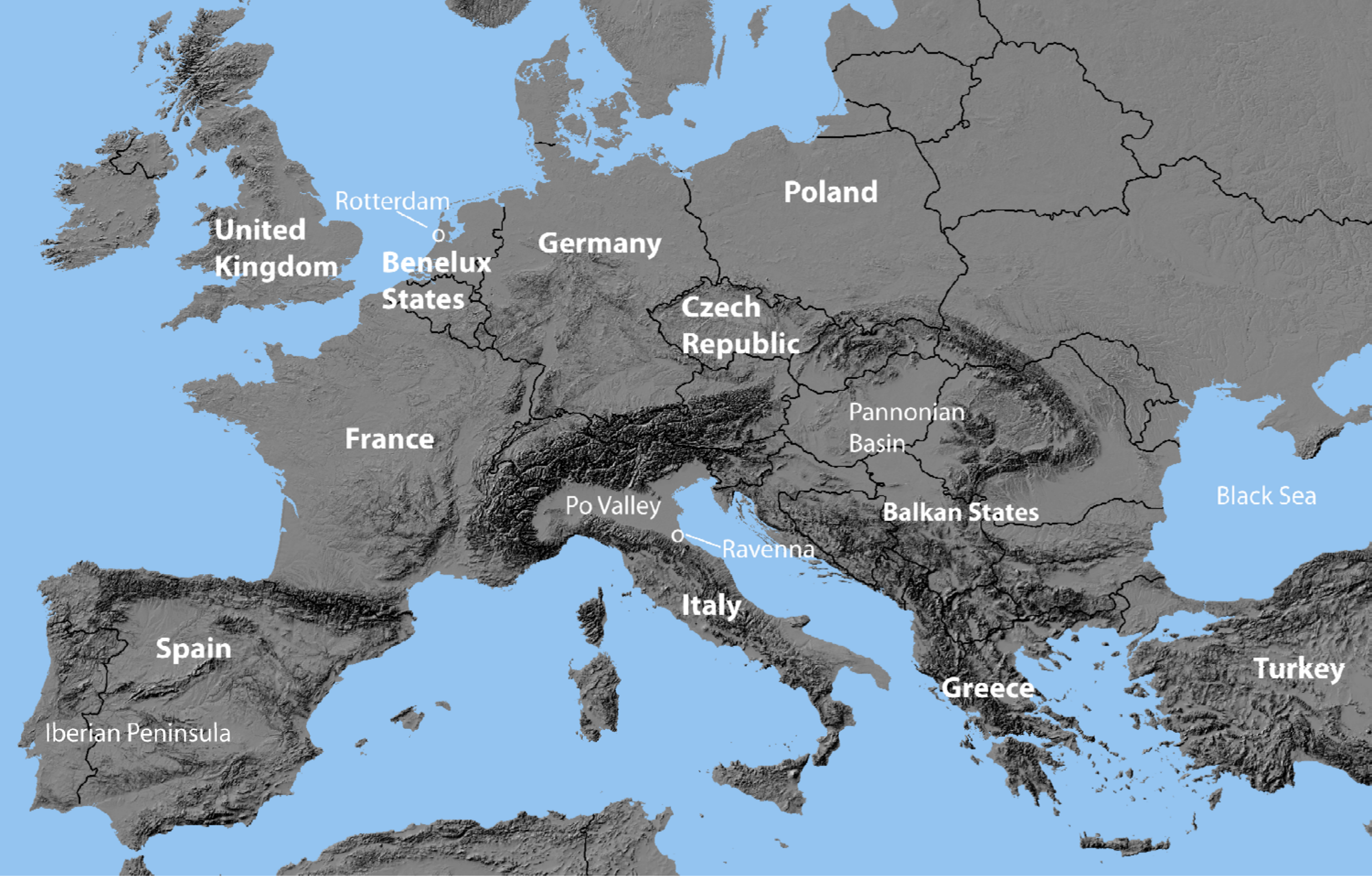

Additional file 3

Climate change effects on Chikungunya transmission in Europe:
Geospatial analysis of vector´s climatic suitability and virus´ temperature requirements

Dominik Fischer, Stephanie M. Thomas, Jonathan E. Suk, Bertrand Sudre, Andrea Hess, Nils B. Tjaden, Carl Beierkuhnlein, Jan C. Semenza
